# Root-Perforation: A New Method of Treatment

**Published:** 1897-06

**Authors:** John Girdwood

**Affiliations:** Edinburgh, Scotland


### International Dental Journal.

Vol. XVIII. June, 1897. No. 6.

### Original Communications.[1]

### ROOT-PERFORATION: A NEW METHOD OF TREATMENT.[2]

BY JOHN GIRDWOOD, D.D.S., L.D.S., EDINBURGH, SCOTLAND.

Among those of our profession to whom modern dentistry is something more than a mere expression, the title of this communication is bound to elicit interest. The man whose daily work is conservation and not substitution, who "crowns" and "bridges" undeterred by anything so long as he has faith in those lines of treatment and in himself, will see in it an attempt to cope with a real difficulty, which will, however, not be so apparent to him whose methods of practice are not strictly modern.

Whether perforation be the result of traumatism or of caries; or be situated in the side of a single root; at the bifurcation of two or more roots; or have travelled down the side of a fang from a supragingival cavity, it is at present deemed a condition so serious as to make the success of any remedial measures very doubtful. If we examine the literature on the subject its meagreness seems to express a certain shyness in handling the treatment of so uncertain

[1] The editor and publishers are not responsible for the views of authors of papers published in this department, nor for any claim to novelty, or otherwise, that may be made by them. No papers will be received for this department that have appeared in any other journal published in the country.

[2] Read before the Academy of Stomatology, Philadelphia, April 27, 1897.

an issue, and what there is of it practically admits that the methods advocated are seldom, if ever, successful.

The varieties of perforation may be roughly divided, according to their causes, into two classes:

*First.* Traumatic, accidental in root-drilling.

*Second.* Carious, as the result of caries.

The traumatic variety is usually deep in the root and small in area, and is the more amenable to treatment if undertaken soon enough to prevent intrusion of the pericementum into the canal or cavity. The second variety generally occurs about the cervix, and is often very extensive. When perforation of either kind occurs it is followed, as indicated above, by intrusion of the soft tissue subjacent—hernia pericementi—and by pain more or less continuous until the sharp edges of the aperture have irritated the intruding soft mass till it becomes inflamed and gangrenous, sloughs, or gives rise to abscess.

The method of treatment most generally followed is that described by Dr. Evans in his book "Crown- and Bridge-Work," and which I shall briefly quote. "Inject the canals with peroxide of hydrogen, then bathe with alcohol, dry with hot air, and fill the canals closely, but not tightly, with cotton saturated with oil of cloves and seal with gutta-percha. Renew the dressing at intervals, preventing the entrance of saliva until the exudation of serum ceases at the perforation, and a film of cicatricial tissue has been formed. Dry the canal thoroughly and fit closely over the perforation a small, flat piece of gutta-percha warmed and applied with a gentle pressure sufficient only to produce adhesion without forcing the gutta-percha through.

"Previously painting the adjacent walls with chloro-gutta-percha will cause the gutta-percha to adhere readily," etc.

While I believe this method may sometimes prove successful where the operation of filling only is required, it is altogether unsuitable in the front teeth when these require to be "crowned." Moreover, the application of a piece of gutta-percha as described is a matter of extreme difficulty, except where the perforation is near the gingival end of a root, and, even when successfully done, the well-known property which gutta-percha possesses of swelling in the presence of moisture is certain, sooner or later, to produce the inflammatory changes leading to abscess of a nature almost incurable, because of the difficulty found in the removal of the cause except by extraction of the tooth. Other methods than the above have been tried, such as shaping a piece of quill, or a section of gold tube to cover the opening, but all of these—which may be summed up as partial intubation—are open to the same objection. They are all, moreover, liable to displacement when a post has to be used in "crowning."

It is obvious, therefore, that for the successful treatment of perforation we require a material essentially different from anything of the kind up till now recommended. It should be sufficiently plastic to adapt itself readily and accurately to the tissues underlying the lesion without danger of displacing them; it must be non-irritant and insoluble, capable of becoming hard and resistant within a reasonable time, and of bearing considerable pressure without fear of movement or fracture. Such a material we possess in copper amalgam. Besides having the qualities already enumerated, copper amalgam is markedly antiseptic, a property of no small value in this connection, while the astringent action of its salts in allaying early inflammation are too well known to need comment here. Its application, which is nothing more or less than entire intubation of the perforated section of the root, is given in detail in the following cases:

My first case of traumatic perforation occurred about six years ago, in a right upper lateral incisor, which I was called upon to crown. The root had been filled some years before with gutta-percha. This I drilled out to what I judged to be about three-fourths the depth of the root, which, at this point, seemed to take a bend to the left. Having reamed out the canal, and fearing it was not quite deep enough to accommodate the pin, I unwisely proceeded to drill the canal a little deeper. I had almost succeeded to my satisfaction, when the patient showed sudden signs of pain, and on withdrawing the drill, wiping out the canal, I found traces of blood. I immediately dried out the canal and carefully dressed it with oil of cloves. Next day the case showed a distinct swelling on the gum over the seat of the perforation, but within three days this swelling had entirely disappeared and all irritation had subsided. At a subsequent appointment I removed the dressing, dried the canal out thoroughly, and proceeded to seal the perforation in the following manner:

Having mixed copper amalgam quite thin I gently carried a small portion of it to the apex with a blunt-pointed instrument rounded at the end,—in fact, an old excavator with the point broken off. The amalgam was gently spread round the upper portion of the canal, a rotatory motion being used to carry it evenly around the interior. No pain was caused by this proceeding, but the patient was just aware of a very slight sensation while it was being done; this sensation subsided when the point was withdrawn. The interior of the canal being thus lined, its depth was not seriously lessened. The patient was now dismissed till the following day, when the amalgam had hardened thoroughly, and at the next sitting a Richmond crown was set, and has done good service up to the present time.

The next is a case of traumatic perforation of a different nature, near the apex of a second upper bicuspid root, in the mouth of a lady for whom I had, four years before, treated an extensive perforation at the bifurcation of a second lower molar, right, now crowned and doing good service.

Miss M. called about fourteen months ago and desired me to crown the left second upper bicuspid.

The tooth had been filled some years previously with copper amalgam by another dentist, and as it had become gradually more and more discolored she determined to have it crowned. I removed the filling and found the pulp alive, I destroyed and removed it with difficulty, the canal being long, flat, and contracted.

This last was then opened up with Gates-Glidden drills and the apex sealed. I now proceeded to enlarge the canal with Peeso's small-sized reamer, which I consider by far the best and safest instrument for this purpose. Having nearly succeeded in enlarging sufficiently for the Logan crown I meant to fit, and when I was using the medium-sized reamer and drilling slowly, the patient showed signs of pain; the drill was at once withdrawn and chips blown out. Suspecting perforation or a near approach to it, I wiped out the canal, but found no sign of blood. I now cautiously explored the whole canal with a stiff, hooked Donaldson bristle and found a tender spot quite close to the end of the root, which I cautiously pressed upon. Immediately the patient showed signs of pain. I now wiped out again and saw this time a tiny drop of serum on the cotton. Having swabbed out the canal with carbolic acid and dried thoroughly, I sealed the perforation with copper amalgam as in the former case. In this case there seems to have been no actual perforation by the reamer, but the wall was so thin that it would no doubt have yielded to the pressure of the cement during the setting of the crown, so that in all probability the actual perforation by the exploring point was the means of avoiding a more serious accident, for had I proceeded with the setting of the crown, there is little doubt that the thinned wall would have been bulged outward, and irritation set up of such a serious kind that successful after-treatment might have been impossible.

In the treatment of carious perforation—the second variety—I have followed similar lines as shown in the following cases:

Mr. H. called about three years ago to have some teeth filled. On examination the left upper lateral crown was missing and the root almost entirely covered by gum. I proposed he should have it crowned, but was informed that this had been twice done about two years before, and that each crown had failed within a few days of its insertion. This had proved so discouraging to the patient that he decided to let it alone. About a year ago he again presented himself and requested an effort should be made to crown.

A thirty-per-cent. solution of cocaine was applied to the hernia-like knuckle of gum occupying the face of the root, and when this had been removed as completely as was deemed possible, a long, funnel-shaped opening was seen. This was washed out, and when bleeding had ceased was packed with cotton dipped in oil of cloves and hydronaphthol and lightly sealed with temporary gutta-percha. In twenty-four hours this dressing was taken out and the walls of the root explored; these were found to be extensively softened and perforated on the left side well below the gum, which projected through the space. At the apex—sealed at previous crownings—about one-eighth of an inch of the normal canal was patent, the remainder being hollowed out as just described. The carious dentine was now cautiously removed by a spoon excavator and the perforation unavoidably enlarged, till, on the left side, it extended from the gum margin towards the apex for fully a third of the root's length, and a new perforation of a like size was made on the right side: thus of the gingival circumference only the lingual and the labial parts remained. This sacrifice, however, brought me to comparatively healthy dentine. The cavity was packed as before and the patient dismissed. At the third visit all traces of decay were removed and the interior of the root roughened for retainage. After careful drying out the soft tissues surrounding the perforation were lightly touched with carbolic acid to prevent ingress of serum and again dried with hot air; copper amalgam was made very thin and plastic, and a little of it gently placed over the perforations and spread over them and their adjacent dentine edges with a round, straight instrument used with a rotatory and rolling motion. In the apex I now placed a smooth iron post, which had been thinly coated with wax,—it was merely heated, plunged in wax, and then cooled,—and round it tamped copper amalgam till the canal was full. Next day this post was heated, the wax melted, and the pin almost dropped out. I then mounted a Richmond crown, which during the three years which have elapsed I have repeatedly seen, and which the patient assures me has never given a moment's trouble or discomfort.

In the spring of 1894, Mrs. M. presented herself at my office. An examination disclosed the right lower first molar abscessed, and with a small polypoid projection of gum growing from the bifurcation of the roots and projecting into the large carious cavity of the crown. Inquiry elicited that the tooth had been crowned some time before she came to me, but the crown had only remained on a short time, and had given rise to almost constant pain till it, fortunately, came off and gave her relief.

Having cleaned out the cavity as thoroughly as possible, I anæsthetized the polypoid portion of gum and cut it off. The decay around the margin of the cavity was thoroughly removed with large spoon-shaped excavators. Free hemorrhage ensued and was checked with hot water as in the other cases. A light dressing of cloves and hydronaphthol was next applied, and the patient dismissed till the following day. After the roots had been treated and filled, the perforation was touched with carbolic acid, thoroughly dried, and soft copper amalgam gently plastered over the whole interior of the cavity, anchorage being got by slight undercutting at the sides. A gold crown was mounted on the root, and has since done excellent work.

In another mouth and about the same time I discovered a type of perforation very commonly met with. The left upper second bicuspid presented an almost hopeless appearance on a cursory examination. The crown was gone and almost the whole of the root surface overgrown by gum, which I got rid of as described above.

Examination then showed a large perforation on the distal side of the root extending from the gingival margin to a point about one-third of the root's length above it. Higher up the pulp was found alive and was destroyed; the apex was sealed and carious dentine removed as far as was deemed safe. A cotton dressing firmly packed now simplified matters, and when the parts had settled into place I tapped the apical end of the root and screwed in a German-siver tube. Between the rough outer surface of this tube and the walls of the root I worked as much copper amalgam as I could safely pack, and in this way restored the shape of the root to the gum-edge. When this had hardened, the projecting portion of the tube was cut off and a porcelain-faced crown mounted in the usual way. This crown is still in position and seems likely to remain.

The above cases all seem to me to point in one direction. They are all types, each varying a little but all common enough to most of us and none of them easy to treat. In other situations the merits of copper amalgam may perhaps be justly disputed, but here they are unquestionably good. The plan I advocate, even if by chance it should not give so uniformly good results as it has done during the last six years, is, I feel convinced, far more reliable than the lines of treatment hitherto adopted. Out of a very large number of cases I cannot record one failure. The result in cases formerly deemed most uncertain or even hopeless has indeed been such, that time and a careful trial of this method seem to me to be alone needed to prove that this question of perforation has been practically solved.

No doubt it will be objected that with due precautions perforation should not occur, but inasmuch as no man can see a root lying in the alveolus, measure its depth or gauge the thickness of its walls, he cannot in every case be expected to cope with abnormality with that degree of certainty which such objectors would imply as possible. In treating a difficulty the mental attitude of expectancy is not everything, we also require the physical sense of touch, and as the conditions imposed by abnormality are often such as to render ordinary precautions of no avail, accidents must happen unless the case be abandoned. Even when an abnormality is successfully diagnosed, the diagnosis does not carry with it perfect results in treatments. This being so, success in the less readily recognizable and quite unrecognizable forms becomes more remote.

If we consider the variations in shape, size, and curvature of certain roots, such as the upper lateral incisors, the upper bicuspids,—particularly the first, often bifid with flattened roots or coalesced canals,—we cannot wonder at an occasional accident even when the dentist is alive to its possible occurrence. Such an accident has, I regret to say, often happened in my hands during the past six years, and in every case the remedy here advocated has succeeded. It is, therefore, with the greatest confidence that I beg to record my experience and to commend its results to my professional confrères.

